# The relationship between visual function and physical performance in the Study of Muscle, Mobility and Aging (SOMMA)

**DOI:** 10.1371/journal.pone.0292079

**Published:** 2023-09-27

**Authors:** Atalie C. Thompson, Eileen Johnson, Michael E. Miller, Jeff D. Williamson, Anne B. Newman, Steve Cummings, Peggy Cawthon, Stephen B. Kritchevsky

**Affiliations:** 1 Department of Ophthalmology, Wake Forest University School of Medicine, Winston Salem, NC, United States of America; 2 Department of Internal Medicine, Section on Gerontology and Geriatric Medicine, Wake Forest University School of Medicine, Winston Salem, NC, United States of America; 3 San Francisco Coordinating Center, California Pacific Medical Center Research Institute, San Francisco, California, United States of America; 4 Division of Public Health Sciences, Wake Forest University School of Medicine, Winston Salem, NC, United States of America; 5 Center for Aging and Population Health, School of Public Health, University of Pittsburgh, Pittsburgh, Pennsylvania; 6 Department of Epidemiology and Biostatistics, University of California, San Francisco, CA, United States of America; University of Rochester, UNITED STATES

## Abstract

**Purpose:**

The relationship of types of visual function to different aspects of physical function, especially strength and coordination, has been understudied, but delineation of these relationships could suggest potentially modifiable targets prior to the onset of disability.

**Methods:**

Cross-sectional analysis of visual function (self-reported eyesight and eye disease, visual acuity, contrast sensitivity) and physical function tests in 877 older adults (mean age 76.36±5.01 years, 59.2% women, and 13.3% Black race). Separate linear regression models were constructed for short physical performance battery (SPPB), expanded SPPB (eSPPB), their components (gait speed, chair stand, balance, narrow walk), stair climb, four-square step, leg extension peak power and strength, and grip strength.

**Results:**

In adjusted models, worse acuity, worse contrast sensitivity, and self-reported poor vision were significantly associated with worse performance on the eSPPB and four-square step test. Worse contrast sensitivity, but not acuity, was significantly associated with shorter balance times, slower chair stand pace, longer stair climb time, and worse SPPB score. Associations of worse acuity and contrast sensitivity with weaker leg extension power, leg strength, and grip strength were attenuated by covariate adjustment. Self-reported macular degeneration, but not cataract or glaucoma, was associated with worse performance on SPPB, eSPPB, balance, stair climb, and four-square step tests in adjusted models. Worse contrast sensitivity and macular degeneration remained associated with worse SPPB and balance after controlling for visual acuity and self-reported eyesight.

**Conclusions:**

Poor contrast sensitivity was more strongly associated with worse physical performance than acuity, especially for complex tasks that dynamically challenge coordination and balance. Future studies should examine if older adults with contrast sensitivity impairment would benefit from targeted intervention to decrease their risk of disability.

## Introduction

Epidemiologic studies have demonstrated that physical function is an important predictor of frailty [1], hospitalizations, and mortality [2, 3]. There has been a great deal of research into neuromuscular and cardiovascular correlates, but there is growing evidence that visual impairment in older adults is also associated with poor physical function and its sequelae–falls [4], hip fractures [5, 6], hospitalizations [7], disability [8], and mortality [9]. Impaired visual acuity is common, affecting more than 3.22 million adults in the U.S., most of whom are >60 years old [10]. Visual acuity measures the smallest letters one can read on a high contrast standardized eye chart (i.e. black letters on a white background). Contrast sensitivity, on the other hand, is the ability to discern differences in shadings and detect outlines of objects. Contrast sensitivity may also decline with age, even in older adults without eye pathology [11], and contrast sensitivity impairment may also be more prevalent than visual acuity impairment [12, 13]. However, contrast sensitivity is not routinely assessed in clinical practice, and the literature relating it to physical function is relatively sparse [12–17].

We recently showed that contrast sensitivity is significantly associated with lower extremity performance on the short physical performance battery (SPPB) and each of its components (gait speed, chair stand pace, and standing balance time) independent of visual acuity, stereoacuity (or the perception of depth when using both eyes), and self-reported visual function in the Health, Aging and Body Composition (ABC) study [18]. This observation suggests that contrast sensitivity is more central to mobility function than more commonly tested measures like visual acuity, but needs to be confirmed. While the basis for the relationship between visual and physical function is unclear, an expanded set of measures may also help to better isolate which specific aspects of physical function are involved, which in turn may provide clues to underlying mechanisms and may even suggest targets for intervention to improve physical function in older adults with visual dysfunction. For example, tests of muscle strength and functional power have not been thoroughly examined in prior studies of visual and physical function. However, it is known that older adults with poor vision are more sedentary [19], which could result in loss of physical strength due to low activity levels. Newer tests that are more challenging to dynamic balance, coordination, and functional power may also help to reveal subclinical or subtle deficits in physical function that are related to visual deficits and which may otherwise not be apparent.

In this analysis, we sought to extend our prior findings using baseline data from the Study of Muscle, Mobility and Aging (SOMMA), which is a new cohort study of adults ≥70 years old residing in Forsyth County, NC and Pittsburgh, PA. SOMMA’s overarching goal is to identify novel determinants of mobility disability. In addition to visual acuity and contrast sensitivity, SOMMA has an expanded set of physical function measures compared to Health ABC and other studies. We hypothesized that lower visual function (visual acuity, contrast sensitivity) and self-reported eye health would be associated with worse performance on both traditional and newer more challenging measures of physical performance and muscle strength. Moreover, given our prior investigation in Health ABC [18], we sought to confirm if contrast sensitivity may be more important to physical performance than visual acuity or self-reported eye health in this contemporary cohort of older adults.

## Materials and methods

### Participants

SOMMA is a prospective cohort study of community-based men and women ≥70 years old. Participants were recruited from April 2019 to December 2021 at two clinical sites–the University of Pittsburgh (Pittsburgh, PA) and Wake Forest University (Winston-Salem, NC). The enrollment and study protocols have been previously described [20]. Potential recruits needed to be willing and able to undergo Magnetic Resonance scans and muscle tissue biopsy to be included. During an initial telephone screening, individuals on anticoagulation therapy were excluded due to risk of bleeding during muscle biopsy. Other exclusions were self-reported inability to climb a flight of stairs or walk ¼ mile, active cancer, or advanced chronic disease such as heart failure, renal failure on dialysis, Parkinson’s disease, and dementia. Those who were initially eligible were invited to an informational screening visit held in-person, by video conference, or over the telephone. Final eligibility for enrollment included demonstration of ability to walk 400 meters at a usual pace at the first day of the baseline visit. In addition, an array of tests of physical, cognitive, and visual function were assessed, as described below. WIRB-Copernicus Group (WCG) Institutional Review Board (WCGIRB, study number 20180764) approved the study as a single IRB. The study was conducted in accordance with Health Insurance Portability and Accountability Act (HIPAA) regulations. All participants provided written informed consent. All data were de-identified for the purpose of analyses.

### Physical function testing

#### Short physical performance battery

The 12-point Short physical performance battery (SPPB) has been extensively used in clinical research and care to assess lower extremity function and is constructed from three sub-categories–gait speed, standing balance, and chair stands [2]. In SOMMA, the standing balance test tested a participant’s ability to maintain static side-by-side, semi-tandem or full-tandem stands for 30 seconds each, thus totaling to 90 seconds. The gait speed (m/sec) was measured over a 4-m walking course. The chair stand pace (stands/sec) was estimated from the time it took the participant to stand up from a seated position and sit down five times without assistance from one’s arms. The performance on each of these components was converted to score from 0–4 based on previously published quartiles of performance, and then summed into a 12-point composite SPPB score where higher scores indicate better performance [2]. The individual measurements for gait speed (m/sec), chair stand pace (stands/sec) and standing balance (0–90 sec) were also evaluated.

In addition, given the higher function of the SOMMA participants at baseline, an expanded SPPB (eSPPB) was measured, which includes the aforementioned tests, as well as a more challenging single-leg balance stand and a 4-meter narrow walk test that requires participants to keep their steps between two parallel lines spaced 20 cm apart [3]. For each component that was not completed successfully, a score of 0 was assigned. Then each of the continuous scores was divided by the maximal performance possible to create a summary score ratio for each component ranging from 0 to 1: the chair stand pace was divided by 1 stand/sec, the gait speed and narrow walk speed by 2 m/s, and the balance test by 90 sec. The final sum of the four component ratios provided an overall eSPPB score ranging from 0 to 4 with higher scores indicating better function [3].

#### Four-square step test

During the four-square step test (FSST) [21], participants were asked to step forward, sideways, and backward into different quadrants to test their dynamic balance and coordination. Three trials were completed with the fastest time (sec) being used in the analysis.

#### Stair climb test

Participants were asked to climb up and down four standard steps (10 inches deep and 6 inches tall) three consecutive times without stopping, and the total time (sec) at the end of the stair climb test was recorded [22]. Participants were permitted to use a handrail if needed.

#### Leg extension strength and power

The Keiser pneumatic resistance device (AIR300 or A420 model; Keiser Sports Health Equipment, Fresno, CA) was used to assess dynamic single leg press power at 1 repetition maximum (1-RM), a measure of leg extension strength, on day 1 of the baseline visit [23]. A 30-minute period of no physical activity followed the 1-RM test; then participants completed 2 trials at each intensity level (40%, 50%, 60%, and 70% of 1-RM) with 30 seconds rest between each trial at the same resistance, and 1 minute rest between each increase in resistance to determine power. While a single leg was used for the leg press, the two plates of the Keiser device were connected when the test was administered. The standardized peak power is the maximum in Watts/kg across the tests at 40–70% of 1 RM from both plates combined.

#### Grip strength

Grip strength [24] was measured with two trials on both the left and right hand using a Jamar dynamometer (Sammons Preston Rolyan, Bolingbrook, IL, USA). The maximum of the right and left grip strength (kg) was used in the analysis.

### Visual function testing

#### Self-reported vision and eye disease

Participants were asked to self-report common age-related eye diseases: cataracts, glaucoma, and macular degeneration (MD). In addition, they were asked to rate the quality of their eyesight on a 6-point scale with 1 being excellent, 2 good, 3 fair, 4 poor, 5 very poor, and 6 completely blind. For the purpose of analysis, this score was subcategorized into a binary variable of poor (“fair, poor, very poor, blind) vs. good (“excellent, good”) eyesight.

#### Bailey-Lovie distance visual acuity

The Bailey-Lovie high contrast distance acuity test was administered at baseline with participants wearing their usual corrective glasses or contact lenses if applicable. The test was administered at 10 feet or 5 feet and a correction was made to the binocular letter count for the non-standard testing distance per Bailey et al. [25, 26]. The corrected letter count was converted to Snellen acuity (Snellen acuity denominator = 20×10^[(55—x)/50]^) and to log minimum angle of resolution (logMAR = 1.1–0.02 * x) where x is the number of letters read correctly after making an adjustment for the testing distance [27]. A decrease in logMAR indicates better visual acuity. The Snellen acuity was further categorized into a clinically relevant cut-off for poor vision (worse than 20/40 vs. 20/40 or better) based on criteria from the American Academy of Ophthalmology [28].

#### Pelli-robson contrast sensitivity

Binocular contrast sensitivity was measured with participants wearing their usual corrective lenses, if applicable, at the standard testing distance of 3 meters (10 feet) for the Pelli-Robson eye chart [29]. The log contrast sensitivity (LCS) was calculated using the conversion (LCS = (0.05*x)-0.15) where x is the number of correct letters read. For the continuous variable, the negative LCS was used in analysis to aid interpretation since lower LCS indicates worse contrast sensitivity. In addition, binary variables were created for moderate impairment or worse (LCS<1.55; ability to read fewer than 34 letters) and severe impairment (LCS≤ 1.3; ability to read 29 letters or fewer) based on thresholds for the Pelli Robson chart used in prior longitudinal cohort studies of adults older than 60 years of age [12, 18, 30]. Such thresholds were selected to help determine whether there is a degree of impairment in contrast sensitivity that might better identify older adults with co-prevalent physical dysfunction.

### Clinical and demographic covariates

Demographic information such as participant age, race, gender, and highest level of education were assessed by questionnaire. Gender was categorized as women and men. Race was categorized into Black and non-Black. Education was categorized as high school or less, some college, college graduate, and post-college work. Participants were asked about a medical history of diabetes mellitus or stroke; and they were considered to have heart disease if they reported a history of heart failure, heart attack or myocardial infarction, blocked artery, aortic stenosis, or atrial fibrillation. Hypertension was determined by a systolic blood pressure of ≥140 mmHg measured at the baseline visit. Body mass index (BMI) was calculated from participant’s measured weight in kilograms divided by height in m^2^. Health behaviors such as alcohol use were assessed by asking for the average number of drinks per week in the past 12 months and smoking status was based on whether they reported never smoking vs. current or past smoking.

### Depressive symptoms

The Center for Epidemiological Studies Depression Scale (CESD-10) is a 10-item Likert scale questionnaire that assesses depressive symptoms in the preceding week [31]. Each individual question is weighted and the summary score ranges from 0–30, with a score greater than or equal to 10 indicating depressive symptoms.

### Statistical methods

Baseline clinical and sociodemographic characteristics of the study participants were stratified by impairment in visual acuity (VA<20/40) or log contrast sensitivity (LCS<1.55), and the association of visual acuity or LCS with each characteristic was assessed using t-tests or Wilcoxon rank-sum for continuous variables and chi-square or Fisher’s exact tests for categorical variables. Next, separate linear regression models were constructed to examine the baseline cross-sectional relationship of visual function and physical function. The following visual function measures were explored as independent variables in separate models: self-reported visual function (Poor vs. Good), logMAR visual acuity, poor visual acuity (worse than 20/40 vs. 20/40 or better), LCS, at least moderately impaired LCS (worse than 1.55 log units vs. 1.55 log units or better), and severely impaired LCS (worse than or equal to 1.3 log units vs. better than 1.3 log units). In addition, self-reported eye diseases (i.e., cataract, glaucoma, macular degeneration) were considered in separate models. The following physical function measures were modeled as the dependent variable in separate models: 1) overall SPPB score 2) each of the individual SPPB components (i.e., gait speed, chair stand pace, and balance time), 3) expanded SPPB score, 4) narrow walk speed, 5) stair climb time, 6) four-square step test time, 7) leg extension standardized peak power, 8) leg extension strength (1-RM) and 9) grip strength.

For each regression of visual function versus physical function, the first analysis was an unadjusted bivariate model (model 1). Next, the model was adjusted for demographic, clinical, and behavioral covariates: age, gender, race, highest level of education, BMI, smoking status, alcohol consumption, hypertension, diabetes mellitus, heart disease, stroke, and depressive mood (CESD-10) (model 2). Since the upper age limit in SOMMA is greater than in Health ABC, the interaction of each vision variable with age was also tested in each model for SPPB and its components to explore if age modified the effect.

To explore the relative contribution of each type of visual function on physical function, separate multivariable models were constructed for each measure of physical function including both performance-based and self-reported vision measures (continuous LCS, logMAR visual acuity, and self-reported vision) without (model 3a) and with adjustment for all covariates (model 3b). Because macular degeneration (MD) was the only self-reported eye disease significantly associated with multiple physical function measures, it was included in a final exploratory multivariable model of multiple visual function variables to determine whether MD explained the association between visual and physical function (model 4). A sensitivity analysis was also performed in multivariable models, removing age to determine the impact of age adjustment. Statistical analyses were conducted in SAS (version 9.4, Cary, NC).

## Results

### Demographic and clinical covariates stratified by visual impairment

Of the 879 participants enrolled in SOMMA at the baseline visit, 877 had completed visual and physical function testing and were included in the analysis. The mean participant age was 76.36±5.01 years, with 59.2% identifying as women and 13.3% as Black race (Table 1). Participants with impairment in VA<20.40 or LCS<1.55 were older and reported higher rates of smoking. LCS<1.55 and VA<20/40 were each associated with self-reported MD but not glaucoma, and only LCS was associated with cataract.

**Table 1:** Association of visual acuity and contrast sensitivity with baseline sociodemographic and clinical variables.

Characteristic		Overall	Visual Acuity			Log Contrast Sensitivity		
			20/40 or better	Worse than 20/40		1.55 or better	Worse than 1.55	
** **		(N = 877)	(N = 445)	(N = 432)	**p-value**	(N = 570)	(N = 304)	**p-value**
**Age, years**		76.4 ± 5.0	75.6 ± 4.3	77.2 ± 5.5	< .0001	75.7 ± 4.7	77.6 ± 5.4	< .0001
**Gender**	**Women**	519 (59.2)	257 (57.8)	262 (60.7)	0.383	345 (60.5)	172 (56.6)	0.258
	**Men**	358 (40.8)	188 (42.3)	170 (39.4)		225 (39.5)	132 (43.4)	
**Race**	**Non-Black**	756 (86.7)	384 (87.1)	372 (86.3)	0.740	495 (87.5)	258 (85.2)	0.341
	**Black**	116 (13.3)	57 (12.9)	59 (13.7)		71 (12.5)	45 (14.9)	
**BMI, kg/m** ^**2**^		27.6 ± 4.6	27.8 ± 4.5	27.4 ± 4.6	0.288	27.7 ± 4.5	27.5 ± 4.7	0.541
**Education**	**High school or less or other**	129 (14.9)	65 (14.8)	64 (15.0)	0.713	83 (14.7)	46 (15.3)	0.241
	**Some college**	202 (23.3)	106 (24.1)	96 (22.4)		144 (25.5)	58 (19.3)	
	**College graduate**	224 (25.8)	118 (26.8)	106 (24.8)		141 (25.0)	82 (27.3)	
	**Post college work**	313 (36.1)	151 (34.3)	162 (37.9)		197 (34.9)	114 (38.0)	
**Smoking status**	**Never**	491 (56.3)	265 (59.8)	226 (52.7)	0.034	338 (59.3)	152 (50.8)	0.017
	**Current or Past**	381 (43.7)	178 (40.2)	203 (47.3)		232 (40.7)	147 (49.2)	
**Alcohol intake, drinks/week, median (IQR)**		0.5 (0, 4)	0.75 (0, 4)	0.25 (0, 4)	0.036	0.5 (0, 4)	0.5 (0, 4)	0.902
**Diabetes Mellitus**		130 (14.9)	60 (13.5)	70 (16.2)	0.263	80 (14.0)	50 (16.6)	0.310
**Hypertension**		246 (28.1)	122 (27.4)	124 (28.7)	0.671	162 (28.4)	83 (27.3)	0.726
**History of heart disease** ^**a**^		97 (11.1)	42 (9.5)	55 (12.8)	0.123	58 (10.2)	39 (13.0)	0.215
** Heart failure**		6 (0.7)	4 (0.9)	2 (0.5)	0.432	3 (0.5)	3 (1.0)	0.425
** Heart attack or myocardial infarction**		23 (2.6)	7 (1.6)	16 (3.7)	0.049	8 (1.4)	15 (5.0)	0.002
** Aortic stenosis**		8 (0.92)	2 (0.5)	6 (1.4)	0.144	5 (0.9)	3 (1.0)	0.861
** Blocked artery**		57 (6.5)	24 (5.4)	33 (7.7)	0.180	32 (5.6)	25 (8.3)	0.127
** Atrial fibrillation**		35 (4.0)	19 (4.3)	16 (3.7)	0.664	23 (4.0)	12 (4.0)	0.972
**Stroke**		21 (2.4)	8 (1.8)	13 (3.0)	0.243	13 (2.3)	7 (2.3)	0.967
**CESD-10 Score**		4.2 ± 3.5	4.2 ± 3.5	4.1 ± 3.5	0.902	4.0 ± 3.5	4.4 ± 3.5	0.092
**Cataracts**		570 (65.4)	277 (62.8)	293 (68.0)	0.109	347 (61.0)	221 (73.7)	0.0002
**Glaucoma**		83 (9.5)	35 (7.9)	48 (11.1)	0.103	47 (8.3)	36 (12.0)	0.076
**Macular degeneration**		69 (7.9)	22 (5.0)	47 (10.9)	0.001	25 (4.4)	44 (14.6)	< .0001

*Note*. Data shown as n(%), mean ± SD. P-values for categorical data from a chi-square test, or Fisher’s exact test if one or more expected counts is <5. P-values for continuous variables from a t-test for normally distributed data, and a Wilcoxon rank-sum test for skewed data. BMI = Body mass index; CESD-10 = Center for Epidemiological Studies Depression Scale

^a^History of heart disease included heart failure, heart attack or myocardial infarction, aortic stenosis, blocked artery, or atrial fibrillation.

### Relationship of visual function and traditional measures of physical performance

**Fig 1:**
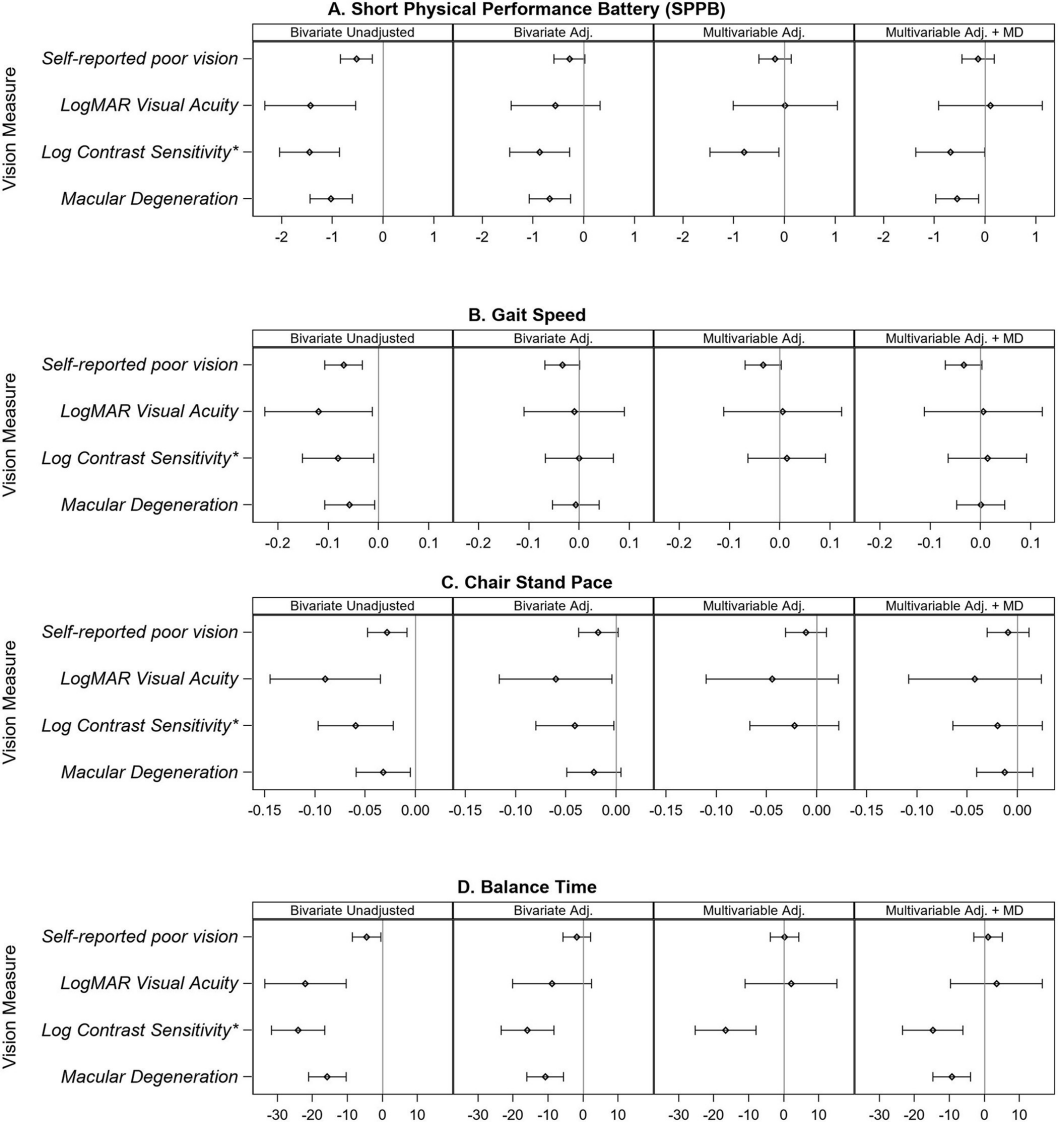
Unadjusted bivariate and adjusted bivariate and multivariable models between multiple vision variables and traditional measures of physical performance. Plots show Beta (95% CI) for physical performance outcomes compared at levels of discrete visual function predictors (self-reported poor vision, macular degeneration) or for a 1-unit difference in continuous predictors (logMAR visual acuity, -Log contrast sensitivity). Panel 1: Bivariate Unadjusted presents the unadjusted association between each measure of visual function and physical performance. Panel 2: Bivariate Adjusted presents the association between each measure of visual function and physical performance, adjusted for clinical and demographic covariates (age, gender, race, education, BMI, smoking status, alcohol consumption, diabetes mellitus, hypertension, heart disease, stroke, CESD-10). Panel 3: Multivariable Adjusted presents the association between multiple measures of visual function (self-reported poor vision, logMAR visual acuity, -log contrast sensitivity) and physical performance adjusted for clinical and demographic covariates (age, gender, race, education, BMI, smoking status, alcohol consumption, diabetes mellitus, hypertension, heart disease, stroke, CESD-10). Panel 4: Multivariable Adjusted + MD presents the association between multiple measures of visual function (self-reported poor vision, logMAR visual acuity, -log contrast sensitivity, plus macular degeneration (MD)) and physical performance, adjusted for clinical and demographic covariates (age, gender, race, education, BMI, smoking status, alcohol consumption, diabetes mellitus, hypertension, heart disease, stroke, CESD-10). The physical performance outcomes are A) short physical performance battery (SPPB) B) gait speed, C) chair stand pace, and D) balance time. *-1 unit log contrast sensitivity.

Fig 1 and S1 Table present the unadjusted and adjusted analysis of vision variables with SPPB and the individual components of gait speed, chair stand pace, and balance time. There was no relationship between SPPB and self-reported poor eyesight, logMAR visual acuity, or VA<20/40 in adjusted models. One unit lower LCS, or being able to read 2.5 lines less, was significantly associated with a 0.87 unit lower SPPB in adjusted analysis (p = 0.004). Both thresholds of severely impaired LCS (≤1.3) and at least moderately impaired LCS (<1.55) were also associated with a lower SPPB in adjusted analyses (S1 Table; S1 Fig).

Worse logMAR VA, lower LCS, and moderately impaired LCS (<1.55) were each associated with slower chair stand pace in adjusted analyses (S1 Fig). Only lower LCS, moderately impaired LCS (<1.55), and severely impaired LCS (≤1.3), but not visual acuity or self-reported eyesight, were associated with shorter balance times in adjusted models. The association between 4-m gait speed and all vision variables was attenuated after adjusting for covariates. There was no significant interaction of age with vision in any of the models for SPPB or its sub-components (data not shown).

### Relationship of visual function with more challenging tests of physical performance

**Fig 2:**
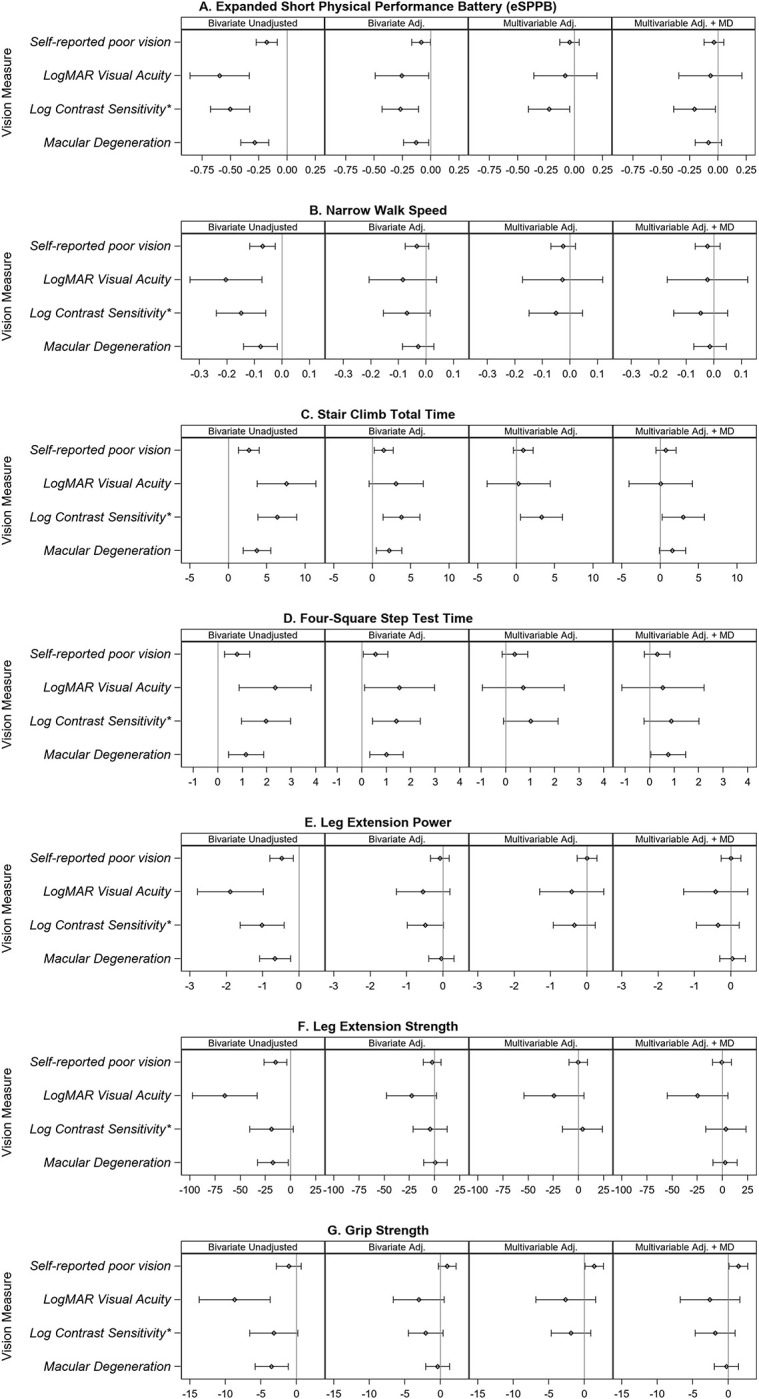
Unadjusted bivariate and adjusted bivariate and multivariable models between multiple vision variables and more challenging tests of physical performance and tests of muscle function. Plots show Beta (95% CI) for physical function outcomes compared at levels of discrete visual function predictors (self-reported poor vision, macular degeneration) or for a 1- unit difference in continuous predictors (logMAR visual acuity, -log contrast sensitivity). Panel 1: Bivariate Unadjusted presents the unadjusted association between each measure of visual function and physical performance or muscle function. Panel 2: Bivariate Adjusted presents the association between each measure of visual function and physical performance or muscle function, adjusted for clinical and demographic covariates (age, gender, race, education, BMI, smoking status, alcohol consumption, diabetes mellitus, hypertension, heart disease, stroke, CESD-10). Panel 3: Multivariable Adjusted presents the association between multiple measures of visual function (self-reported poor vision, logMAR visual acuity, -log contrast sensitivity) and physical performance or muscle function, adjusted for clinical and demographic covariates (age, gender, race, education, BMI, smoking status, alcohol consumption, diabetes mellitus, hypertension, heart disease, stroke, CESD-10). Panel 4: Multivariable Adjusted + MD presents the association between multiple measures of visual function (self-reported poor vision, logMAR visual acuity, -log contrast sensitivity, plus macular degeneration (MD)) and physical performance or muscle function, adjusted for clinical and demographic covariates (age, gender, race, education, BMI, smoking status, alcohol consumption, diabetes mellitus, hypertension, heart disease, stroke, CESD-10). The physical performance and muscle function outcomes are A) expanded short physical performance battery (eSPPB) B) narrow walk speed, C) stair climb time, D) four-square step test time, E) leg extension peak power, F) leg extension strength, and G) grip strength. *-1 unit log contrast sensitivity.

Findings for the expanded SPPB were similar to those of the SPPB (Table 2), but the relationships were more robust to full adjustment, with associations remaining between worse performance on the eSPPB and self-reported poor vision, worse logMAR visual acuity, worse LCS, LCS<1.5, and LCS≤1.3. Worse logMAR VA, VA worse than 20/40, worse LCS, moderately impaired LCS (<1.55), and severely impaired LCS (≤1.3) were each associated with slower narrow walk pace and slower performance (i.e. longer times) on the four-square step test (FSST) in adjusted models (Table 2; Fig 2; S2 Fig). Only moderately and severely impaired LCS remained associated with slower narrow walk in adjusted models. Also, slower performance on the FSST remained associated with self-reported poor vision, worse logMAR visual acuity, worse LCS, and severely impaired LCS in adjusted models.

**Table 2:** Association of individual vision variables with more challenging measures of physical performance.

		Expanded SPPB	Narrow Walk Speed (m/sec)	Stair Climb (sec)	Four-Square Step Test (sec)
		Beta (95% CI), p-value	Beta (95% CI), p-value	Beta (95% CI), p-value	Beta (95% CI), p-value
**Self-reported poor vs. better vision**	Model 1	-0.18 (-0.27, -0.09), p<0.001*	-0.07 (-0.12, -0.03), p = 0.003*	2.67 (1.32, 4.02), p<0.001*	0.78 (0.26, 1.3), p = 0.003*
	Model 2	-0.09 (-0.17, 0), p = 0.042*^+^	-0.03 (-0.08, 0.01), p = 0.135	1.48 (0.24, 2.71), p = 0.02*^+^	0.56 (0.06, 1.06), p = 0.029*^+^
**LogMAR visual acuity**	Model 1	-0.6 (-0.86, -0.34), p<0.001*	-0.2 (-0.33, -0.07), p = 0.002*	7.57 (3.76, 11.38), p<0.001*	2.34 (0.87, 3.81), p = 0.002*
	Model 2	-0.26 (-0.49, -0.02), p = 0.036*^+^	-0.08 (-0.21, 0.04), p = 0.181^+^	3.09 (-0.45, 6.63), p = 0.087^+^	1.54 (0.11, 2.96), p = 0.035*^+^
**Visual acuity worse than 20/40**	Model 1	-0.11 (-0.17, -0.04), p = 0.002*	-0.04 (-0.08, -0.01), p = 0.009*	0.88 (-0.1, 1.86), p = 0.077	0.4 (0.03, 0.77), p = 0.035*
	Model 2	-0.05 (-0.11, 0.01), p = 0.123^+^	-0.02 (-0.05, 0.01), p = 0.157^+^	-0.01 (-0.9, 0.88), p = 0.975^+^	0.28 (-0.08, 0.63), p = 0.128^+^
**Log Contrast Sensitivity** ^**a**^	Model 1	-0.5 (-0.68, -0.33), p<0.001*	-0.15 (-0.24, -0.06), p = 0.001*	6.36 (3.82, 8.9), p<0.001*	1.97 (0.96, 2.97), p<0.001*
	Model 2	-0.27 (-0.43, -0.11), p<0.001**^+^	-0.07 (-0.15, 0.01), p = 0.107^+^	3.79 (1.41, 6.18), p = 0.002*^+^	1.4 (0.43, 2.38), p = 0.005*^+^
**Log Contrast Sensitivity <1.5 (Moderate to severe impairment)**	Model 1	-0.17 (-0.24, -0.1), p<0.001*	-0.07 (-0.1, -0.04), p<0.001*	1.76 (0.73, 2.78), p<0.001*	0.55 (0.15, 0.94), p = 0.006*
	Model 2	-0.11 (-0.17, -0.05), p<0.001*^+^	-0.05 (-0.08, -0.01), p = 0.005*^+^	1.02 (0.09, 1.96), p = 0.033*^+^	0.34 (-0.04, 0.72), p = 0.084^+^
**Log contrast sensitivity ≤1.3 (Severe impairment)**	Model 1	-0.27 (-0.4, -0.14), p<0.001*	-0.08 (-0.15, -0.02), p = 0.017*	4.62 (2.69, 6.55), p<0.001*	1.34 (0.58, 2.09), p<0.001*
	Model 2	-0.16 (-0.27, -0.04), p = 0.008*^+^	-0.05 (-0.11, 0.01), p = 0.103^+^	3.38 (1.64, 5.12), p<0.001*^+^	1.08 (0.36, 1.8), p = 0.004*^+^
**Macular degeneration**	Model 1	-0.29 (-0.41, -0.16), p<0.001*	-0.08 (-0.14, -0.02), p = 0.012*	3.72 (1.91, 5.53), p<0.001*	1.15 (0.43, 1.87), p = 0.002*
	Model 2	-0.13 (-0.24, -0.02), p = 0.022*^+^	-0.03 (-0.09, 0.03), p = 0.331^+^	2.17 (0.52, 3.83), p = 0.01*^+^	1.01 (0.32, 1.69), p = 0.004*^+^

*Note*. Model 1 is unadjusted bivariate analysis. Model 2 is adjusted for age, gender, race, education, body mass index, smoking status, alcohol consumption, diabetes mellitus, hypertension, heart disease, stroke, CESD-10.

LogMAR = logarithm of the minimum angle of resolution.

^a^Coefficients are for a 1 unit lower log contrast sensitivity (-LCS)

*P-value is <0.05

^+^P-value is <0.05 if age is removed from the model.

Lower LCS, moderately impaired LCS (<1.55), severely impaired LCS (≤1.3), and self-reported poor eyesight were each significantly associated with worse performance (i.e. longer times) on the stair climb test in unadjusted and adjusted models. The total stair climb time was not associated with visual acuity in adjusted models.

### Relationship of visual function with tests of muscle function

Table 3 presents the unadjusted and adjusted analysis of the vision variables with tests of muscle function which included leg extension peak power, leg extension strength (1-RM), and grip strength. Worse logMAR VA and VA<20/40 were each associated with weaker grip strength and weaker leg power in unadjusted analyses (Fig 2; S3 Fig). However, self-reported eyesight was not associated with grip strength. Adjustment for covariates attenuated the association between visual dysfunction and tests of muscle function. Only severely impaired LCS (≤1.3) remained associated with weaker grip strength following adjustment.

**Table 3:** Association of individual vision variables with tests of muscle function.

		Leg extension standardized peak power (40–70% of 1 Repetition Maximum, Watts/kg)	Leg extension strength (1 Repetition Maximum, Watts/Kg)	Grip strength (kg)
		Beta (95% CI), p-value	Beta (95% CI), p-value	Beta (95% CI), p-value
**Self-reported poor vs. better vision**	Model 1	-0.48 (-0.8, -0.16), p = 0.004*	-15.06 (-26.47, -3.65), p = 0.01*	-1.1 (-2.86, 0.66), p = 0.222
	Model 2	-0.09 (-0.35, 0.17), p = 0.513	-2.31 (-11.1, 6.48), p = 0.606	0.93 (-0.32, 2.17), p = 0.144
**LogMAR visual acuity**	Model 1	-1.89 (-2.8, -0.99), p<0.001*	-65.42 (-97.49, -33.34), p<0.001*	-8.72 (-13.72, -3.73), p<0.001*
	Model 2	-0.55 (-1.29, 0.19), p = 0.144^+^	-22.92 (-47.88, 2.04), p = 0.072^+^	-3.06 (-6.64, 0.52), p = 0.094^+^
**Visual acuity worse than 20/40**	Model 1	-0.32 (-0.55, -0.09), p = 0.006*	-9.83 (-17.98, -1.68), p = 0.018*	-1.77 (-3.04, -0.51), p = 0.006*
	Model 2	-0.12 (-0.31, 0.06), p = 0.19^+^	-2.29 (-8.53, 3.95), p = 0.472	-0.76 (-1.65, 0.13), p = 0.096^+^
**Log Contrast Sensitivity** ^**a**^	Model 1	-1.02 (-1.62, -0.41), p = 0.001*	-18.98 (-40.64, 2.68), p = 0.086	-3.21 (-6.58, 0.16), p = 0.062
	Model 2	-0.49 (-0.99, 0.01), p = 0.055^+^	-4.52 (-21.44, 12.4), p = 0.601^+^	-2.09 (-4.51, 0.34), p = 0.093^+^
**Log Contrast Sensitivity <1.5 (Moderate to severe impairment)**	Model 1	-0.26 (-0.5, -0.01), p = 0.039*	-5.97 (-14.59, 2.64), p = 0.174	-0.79 (-2.13, 0.54), p = 0.244
	Model 2	-0.15 (-0.34, 0.05), p = 0.137^+^	-3.39 (-9.99, 3.2), p = 0.313^+^	-0.74 (-1.68, 0.2), p = 0.125^+^
**Log contrast sensitivity ≤1.3 (Severe impairment)**	Model 1	-0.48 (-0.93, -0.03), p = 0.037*	-8.26 (-24.27, 7.75), p = 0.312	-1.56 (-4.05, 0.93), p = 0.22
	Model 2	-0.28 (-0.64, 0.07), p = 0.118^+^	-6.97 (-19.06, 5.13), p = 0.259^+^	-1.97 (-3.7, -0.24). p = 0.026*^+^
**Macular degeneration**	Model 1	-0.66 (-1.09, -0.24), p = 0.002*	-17.6 (-32.77, -2.43), p = 0.023*	-3.51 (-5.85, -1.17), p = 0.003*
	Model 2	-0.04 (-0.39, 0.3), p = 0.801	0.76 (-10.89, 12.41), p = 0.898	-0.4 (-2.06, 1.26), p = 0.638

*Note*. Model 1 is unadjusted bivariate analysis. Model 2 is adjusted for age, gender, race, education, body mass index, smoking status, alcohol consumption, diabetes mellitus, hypertension, heart disease, stroke, CESD-10.

LogMAR = logarithm of the minimum angle of resolution.

^a^Coefficients are for a 1 unit lower log contrast sensitivity (-LCS)

*P-value is <0.05

^+^P-value is <0.05 if age is removed from the model.

### Relationship of self-reported eye disease with physical function

Self-reported eye diseases, such as cataract, glaucoma, or macular degeneration, were also examined with the above outcomes. While self-reported cataract and glaucoma did not have significant associations with physical function (data not shown), macular degeneration (MD) was associated with multiple worse performance on multiple physical function outcomes in adjusted models, including SPPB, eSPPB, balance, FSST, and stair climb (S1 Table, Tables 2 and 3). Thus, macular degeneration was considered in further exploratory analyses with other vision variables.

### Relationship of multiple vision variables with physical performance and muscle function

Multiple vision variables were considered simultaneously in exploratory multivariable analyses to determine which vision variable may be more important to certain aspects of physical performance or muscle function (model 3) (S2–S4 Tables). LCS remained significantly and independently associated with SPPB even when including logMAR visual acuity, self-reported eyesight and other covariates in the model (p = 0.022). Next, macular degeneration was included in the model to explore if the relationship of MD with physical performance was explained by visual function; and both LCS (p = 0.048) and MD (p = 0.01) remained significantly associated with SPPB independent of other vision variables and covariates (model 4) (Fig 1; S2 Table). Similar results for LCS were demonstrated in models where eSPPB, balance time, and total stair climb time were the outcome and multiple vision variables and covariates were included (model 3b) (Fig 2; S3 Table). However, if MD was included in the model, only LCS was associated with eSPPB and stair climb time, and only MD was associated with FSST independent of all other vision variables and covariates (model 4).

## Discussion

Our results indicate that contrast sensitivity, which is not typically assessed as part of routine ophthalmologic or primary care exams, may be more important to physical dysfunction than visual acuity [18]. Also, newer tests that were more challenging, especially to dynamic balance and coordination, elicited more dysfunction than traditional 4-m gait speed. This observation may suggest that dysfunction in balance could be an earlier finding that occurs before more overt gait dysfunction, though longitudinal studies are needed to confirm this hypothesis. Also, these results further extend prior findings by showing that tests of functional power, such as the stair climb test, were associated with visual dysfunction. Moreover, while higher levels of vision impairment are known to impact gait and balance, our findings indicate that self-reported ophthalmic disease may not necessarily be sufficient to explain the relationship between poor vision and worse physical function, and that the association of physical function with age-related retinal pathology may not be fully explained by tests of vision. These latter results raise the hypothesis that there may be a shared pathophysiologic pathway between visual and physical function.

There are numerous studies linking poor vision to self-reported or performance-based assessments of physical function and activities of daily living [12, 13, 32, 33], but the nuanced role of particular aspects of vision and mobility and the pathways linking them are less well understood. This study re-demonstrated a strong and significant relationship between poor visual function and poor performance on SPPB which complements similar findings in Health ABC [18] and in a recent 2-community based study by Guo et al. [13]. Moreover, LCS was the only visual function measure that remained significantly associated with multiple measures of physical performance, independent of visual acuity or self-reported vision and other comorbidities. This finding corroborates what we recently observed in Health ABC, where LCS was associated with SPPB and its components of gait speed, balance, and chair pace, independent of visual acuity, stereoacuity, and self-reported vision [18]. Despite these similar findings between SOMMA and Health ABC, there were some key differences in the results when we examined the individual components of the SPPB, which likely reflect the underlying characteristics of the participant population. Health ABC was a more heterogeneous population with ~40% non-white and the participants were approximately 73–83 years of age at the year 3 visit, while ~87% of participants in SOMMA self-identified as white and there was a larger age range at baseline (70–94 years). Although age did not modify the relationship of vision with SPPB in SOMMA, it is possible that the difference in age range between SOMMA and Health ABC explains some of the differences in findings. Moreover, unlike the Health ABC participants, those in SOMMA did not demonstrate a strong persistent relationship between poor visual function and the gait speed component of SPPB in adjusted analyses. Rather, there was a significant and persistent relationship of poor contrast sensitivity with longer balance times and worse performance on more challenging tests that unmasked deficits in dynamic balance and coordination, such as the narrow walk and 4-square step test.

Postural stability is known to decline in older adults over time [34, 35], and risk factors for the development of postural instability are important to identify since such instability can contribute to gait dysfunction and fall risk [12]. Contrast sensitivity is more important for pattern recognition and depth perception, which may explain the pivotal role we observe with respect to balance in particular. The literature also suggests that poor contrast sensitivity may be associated with frailty, which could be related to mobility outcomes [36–38]. In common age-related eye diseases, like glaucoma and age-related macular degeneration, contrast sensitivity deficits can also occur earlier in the disease process, before more overt visual acuity deficits [11]. Thus, it is possible that distance visual acuity may have played less of a role in SOMMA and Health ABC than LCS because the visual deficits were relatively mild which may suggest a milder stage of underlying eye disease or age-related visual dysfunction.

The persistent association of LCS across tests of balance and coordination rather than 4-m gait speed also raises the hypothesis that LCS may be associated with preclinical balance deficits that may precede more overt gait dysfunction. For example, the narrow walk test adds an additional dynamic balance challenge to gait and was designed for use in populations with greater functional reserve to uncover more subtle subclinical mobility dysfunction [3]. The association of LCS with narrow walk gait speed was stronger than traditional 4-m gait speed, and more robust to covariate adjustment. Similarly, there was a significant association of worse LCS with worse performance on the four-square step test, which is a test of dynamic balance and coordination that has been well-validated for use in older adults [39]. Longer FSST times have been associated with increased fall risk [39] and postural instability in a number of neurologic diseases including early Parkinson’s disease [40, 41], chronic stroke [42], and multiple sclerosis [43]. However, to our knowledge this is the first study to examine the relationship of visual function with performance on the FSST. The FSST is also a cognitively demanding locomotor task. By requiring participants to remember and execute a series of steps, the FSST adds a complex challenge to cognitive-motor control and thus may also uncover subclinical impairment in executive function [41]. Thus, the association of visual dysfunction with worse performance on the FSST may not only indicate a deficit in physical function but also could suggest mild cognitive dysfunction. Numerous studies have shown an association between visual impairment and poor cognitive performance on cognitive assessments as well as future cognitive decline [44–46], but our findings may also suggest that early visual dysfunction in LCS could be a biomarker of subclinical cognitive dysfunction. We have also observed similar relationships in the Brain Networks and Mobility Study where cognitively unimpaired older adults with poor LCS but normal visual acuity demonstrated worse postural stability when challenged to stand on a foam surface [47]. Cognitive reserve, which is a latent construct describing one’s ability to use cognitive processing to adapt and optimize performance in the setting of brain injury or stress, has also been shown to be protective against mobility disability [48, 49]. Consequently, lower cognitive reserve may place one at risk of mobility dysfunction. Given that the deficits observed in balance and coordination were unmasked by tasks that challenged both physical and cognitive function, we suspect that these observations may be subtle signs of dysfunction that could be occurring earlier in a process of age-related physical decline. Ongoing longitudinal investigation will help determine if these assessments may identify a cohort of older adults with visual impairment and subclinical deficits in balance, coordination, and cognition that contribute to risk of falls and mobility disability.

Another unique aspect of SOMMA is the incorporation of tests of muscle strength and power. Adults with visual impairment have been shown to have more sedentary behavior [19] as well as lower physical activity participation if they have lower self-efficacy beliefs [50]. Grip strength has also been proposed as a general biomarker of aging because it has been associated with not only general strength, but also numerous other measures of mobility function as well as downstream adverse outcomes such as falls, fractures, multi-morbidity, cognitive impairment, and mortality [51]. Thus, we expected participants to have not only weaker lower extremity muscle strength and power but also weaker grip strength. However, we observed that associations were more variable across vision and muscle measures and were attenuated by covariate adjustment. Only severely impaired LCS remained significantly associated with grip strength. Although poor vision was not independently associated with these tests of muscle strength or power, we did observe that poor LCS was associated with worse performance on both traditional and newer tests of functional leg power such as the chair stand pace and stair climb, respectively. This study is the first to examine the impact of vision on the stair climb test, and mechanisms related to coordination and power as well as functional reserve are likely involved. The stair climb test [22] requires dynamic coordination and challenges both functional power and reserve. Longer stair climb times were evident in those with lower visual function. These findings were remarkable given the performance-based visual deficits in visual acuity and LCS in this population were relatively mild to moderate and the fact that self-reported visual function was not associated with chair pace or leg power on the Keiser leg press. This finding may suggest that even subclinical visual dysfunction that participants do not perceive could subconsciously contribute to lower functional power when performing a more complex activity that also challenges coordination and functional reserve such as climbing stairs.

Another intriguing finding was the significant and persistent contribution of both LCS and macular degeneration to multiple physical function measures independent of each other, other vision measures, and many other clinical and demographic covariates. Although contrast sensitivity is known to be affected in the most prevalent age-related eye diseases, dysfunction in contrast sensitivity may also occur in normal aging in the absence of discrete pathology [11]. Thus, the fact that the relationship of physical function with contrast sensitivity was not fully explained by self-reported eye disease may be due to the presence of unknown eye disease or other age-related visual changes that are not overtly pathologic. More surprising was the finding that macular degeneration was associated with these same measures of balance and mobility independent of performance-based or self-reported visual function. These associations were robust to full adjustment for covariates in the models of SPPB and balance. The association of contrast sensitivity impairment with diverse pathologic and non-pathologic age-related processes may suggest a common underlying biochemical mechanism that is not directly measured by tests of vision. One possibility is that shared neurodegenerative pathways in the brain may impair contrast sensitivity and physical function, and could be reflected in the retina as an extension of the brain. Testing for contrast sensitivity is inexpensive and quick using standard eye charts, but it is not currently measured in clinical practice [11]. Future studies should consider whether assessment of contrast sensitivity may be useful to incorporate into clinical settings and should be studied in relationship to brain function.

### Strengths and limitations

Our study has several notable strengths. Given the large sample size, we were able to include ample adjustment for multiple demographic and clinical covariates that could be potential a priori confounders, though residual unmeasured confounding could exist. We also explored whether the relative contribution of any visual function measure was more important to physical function and we reconfirmed that LCS remained significantly associated with multiple physical function measures independent of visual acuity or self-reported visual function. Moreover, we explored whether self-reported eye disease may explain the relationship between vision and physical function. This led to an unexpected but intriguing finding wherein both poor LCS and macular degeneration remained independent of each other and other covariates and vision measures in several models of physical function. Such relationships may hint at undiscovered shared pathways between contrast sensitivity, macular degeneration and physical mobility. The frequency of self-reported eye disease did not allow for a meaningful sensitivity analysis of the relationship of vision and physical function excluding self-reported eye diseases from the analyses, but this should be done in other larger cohorts to determine the role of visual dysfunction due to normal aging.

The study also has some limitations. This was a cross-sectional analysis of the baseline dataset and so risk and causality cannot be surmised. Only presenting corrected binocular distance visual acuity and contrast sensitivity were tested, wherein participants wore their corrective lenses but were not refracted. Moreover, the participants did not undergo ophthalmic examination to confirm self-reported eye diagnoses, and prior ophthalmic surgery was not assessed. It is possible that the remaining visual dysfunction is related to previously undiagnosed eye pathology or uncorrected residual refractive error.

## Conclusions

In this highly functioning cohort of older adults, we have confirmed a significant cross-sectional relationship between visual function and lower extremity physical function, which was most prominent in more challenging tests of dynamic balance and coordination. Such findings may be early or subclinical signs of gait dysfunction. We have also confirmed a primary role of contrast sensitivity, rather than visual acuity, in relationship to physical performance. Whether contrast sensitivity, which is not currently assessed in clinical settings, could be used to identify older adults at risk of mobility dysfunction is yet to be determined.

## Supporting information

S1 Table Association of individual vision variables with traditional measures of physical performance. (DOCX)

S2 Table Multivariable association of multiple vision variables with traditional measures of physical performance. (DOCX)

S3 Table Multivariable association of multiple vision variables with more challenging tests of physical performance. (DOCX)

S4 Table Multivariable association of multiple vision variables with tests of muscle function. (DOCX)

S1 Fig Unadjusted bivariate and adjusted bivariate models between vision variables and traditional measures of physical performance. Plots show Beta (95% CI) for physical performance outcomes compared at levels of discrete visual function predictors (visual acuity <20/40, log contrast sensitivity < 1.55, or log contrast sensitivity ≤1.3). Panel 1: Bivariate Unadjusted presents the unadjusted association between each measure of visual function and physical performance. Panel 2: Bivariate Adjusted presents the association between each measure of visual function and physical performance, adjusted for clinical and demographic covariates (age, gender, race, education, BMI, smoking status, alcohol consumption, diabetes mellitus, hypertension, heart disease, stroke, CESD-10). The physical performance outcomes are A) short physical performance battery (SPPB) B) 4-m gait speed, C) chair stand pace, and D) balance time.
(TIF)

S2 Fig Unadjusted bivariate and adjusted bivariate models between vision variables and more challenging tests of physical performance. Plots show Beta (95% CI) for physical function outcomes compared at levels of discrete visual function predictors (visual acuity <20/40, log contrast sensitivity < 1.55, or log contrast sensitivity ≤1.3). Panel 1: Bivariate Unadjusted presents the unadjusted association between each measure of visual function and physical performance or muscle function. Panel 2: Bivariate Adjusted presents the association between each measure of visual function and physical performance or muscle function, adjusted for clinical and demographic covariates (age, gender, race, education, BMI, smoking status, alcohol consumption, diabetes mellitus, hypertension, heart disease, stroke, CESD-10). The physical performance or muscle function outcomes are A) expanded short physical performance battery (eSPPB) B) narrow walk speed, C) stair climb time, D) four-square step test time.
(TIF)

S3 Fig Unadjusted bivariate and adjusted bivariate models between vision variables and tests of muscle function. Plots show Beta (95% CI) for muscle function outcomes compared at levels of discrete visual function predictors (visual acuity <20/40, log contrast sensitivity < 1.55, or log contrast sensitivity ≤1.3). Panel 1: Bivariate Unadjusted presents the unadjusted association between each measure of visual function and muscle function. Panel 2: Bivariate Adjusted presents the association between each measure of visual function and muscle function, adjusted for clinical and demographic covariates (age, gender, race, education, BMI, smoking status, alcohol consumption, diabetes mellitus, hypertension, heart disease, stroke, CESD-10). The muscle function outcomes are A) leg extension peak power, B) leg extension strength, and C) grip strength.
(TIF)
