# Supplementary material for: The relationship between visual function and physical performance in the Study of Muscle, Mobility and Aging (SOMMA)
Source: PLoS One. 2023 Sep 27;18(9):e0292079. doi: 10.1371/journal.pone.0292079 (PMC10529600; doi:10.1371/journal.pone.0292079)
Supplement: S1 Table — (DOCX) [file pone.0292079.s001.docx]

|  |  | **SPPB** | **4-Meter Gait Speed (m/sec)** | **Chair Stand Pace (stands/sec)** | **Balance Time (sec)** |
| --- | --- | --- | --- | --- | --- |
|  |  | Beta (95% CI), p-value | Beta (95% CI), p-value | Beta (95% CI), p-value | Beta (95% CI), p-value |
| **Self-reported poor vs. better vision** | Model 1 | -0.53 (-0.84, -0.21), p=0.001* | -0.07 (-0.11, -0.03), p<0.001* | -0.03 (-0.05, -0.01), p=0.005* | -4.55 (-8.64, -0.45), p=0.03* |
|  | Model 2 | -0.28 (-0.59, 0.02), p=0.068^+^ | -0.03 (-0.07, 0), p=0.054^+^ | -0.02 (-0.04, 0), p=0.078^+^ | -1.81 (-5.72, 2.11), p=0.366 |
| **LogMAR visual acuity** | Model 1 | -1.44 (-2.33, -0.54), p=0.002* | -0.12 (-0.23, -0.01), p=0.028* | -0.09 (-0.14, -0.03), p=0.001* | -22.01 (-33.61, -10.41), p<0.001* |
|  | Model 2 | -0.56 (-1.44, 0.32), p=0.213^+^ | -0.01 (-0.11, 0.09), p=0.839 | -0.06 (-0.12, 0), p=0.036*^+^ | -8.94 (-20.21, 2.33), p=0.121^+^ |
| **Visual acuity worse than 20/40** | Model 1 | -0.22 (-0.45, 0), p=0.055 | -0.02 (-0.04, 0.01), p=0.211 | -0.01 (-0.03, 0), p=0.047* | -4.24 (-7.17, -1.3), p=0.005* |
|  | Model 2 | -0.08 (-0.3, 0.13), p=0.45 | 0 (-0.02, 0.03), p=0.9 | -0.01 (-0.02, 0), p=0.17^+^ | -2.12 (-4.93, 0.69), p=0.14^+^ |
| **Log Contrast Sensitivity^a^** | Model 1 | -1.45 (-2.05, -0.86), p<0.001* | -0.08 (-0.15, -0.01), p=0.027* | -0.06 (-0.1, -0.02), p=0.002* | -24.09 (-31.74, -16.45), p<0.001* |
|  | Model 2 | -0.87 (-1.46, -0.28), p=0.004*^+^ | 0 (-0.07, 0.07), p=0.994 | -0.04 (-0.08, 0), p=0.038*^+^ | -15.91 (-23.47, -8.36), p<0.001*^+^ |
| **Log Contrast Sensitivity <1.5 (Moderate to severe impairment)** | Model 1 | -0.42 (-0.66, -0.18), p<0.001* | -0.03 (-0.06, 0), p=0.029* | -0.03 (-0.04, -0.01), p<0.001* | -6.75 (-9.82, -3.68), p<0.001* |
|  | Model 2 | -0.27 (-0.5, -0.04), p=0.022*^+^ | -0.01 (-0.03, 0.02), p=0.53^+^ | -0.02 (-0.04, -0.01), p=0.004*^+^ | -4.73 (-7.69, -1.77), p<0.001*^+^ |
| **Log contrast sensitivity** $\boldsymbol{\leq}$**1.3 (Severe impairment)** | Model 1 | -0.71 (-1.16, -0.27), p=0.002* | -0.03 (-0.09, 0.02), p=0.224 | -0.02 (-0.05, 0.01), p=0.181 | -10.65 (-16.45, -4.85), p<0.001* |
|  | Model 2 | -0.43 (-0.86, -0.01), p=0.047*^+^ | 0 (-0.04, 0.05), p=0.848 | -0.01 (-0.04, 0.02), p=0.491 | -6.87 (-12.36, -1.37), p<0.001*^+^ |
| **Macular degeneration** | Model 1 | -1.03 (-1.45, -0.61), p<0.001* | -0.06 (-0.11, -0.01), p=0.024* | -0.03 (-0.06, -0.01), p=0.019* | -15.8 (-21.16, -10.43), p<0.001* |
|  | Model 2 | -0.67 (-1.08, -0.27), p=0.001*^+^ | -0.01 (-0.05, 0.04), p=0.774 | -0.02 (-0.05, 0), p=0.108^+^ | -10.84 (-16.03, -5.65), p<0.001*^+^ |

*Note*. Model 1 is unadjusted bivariate analysis. Model 2 is adjusted for age, gender, race, education, body mass index, smoking status, alcohol consumption, diabetes mellitus, hypertension, heart disease, stroke, CESD-10. LogMAR = logarithm of the minimum angle of resolution.

^a^Coefficients are for a 1 unit lower log contrast sensitivity (-LCS).

*P-value is <0.05. ^+^P-value is <0.05 if age is removed from the model.
