# Supplementary material for: The relationship between visual function and physical performance in the Study of Muscle, Mobility and Aging (SOMMA)
Source: PLoS One. 2023 Sep 27;18(9):e0292079. doi: 10.1371/journal.pone.0292079 (PMC10529600; doi:10.1371/journal.pone.0292079)
Supplement: S2 Table — (DOCX) [file pone.0292079.s002.docx]

|  |  | **SPPB Score** | **4-Meter Gait Speed (m/sec)** | **Chair Stand Pace (stands/sec)** | **Balance Time (sec)** |
| --- | --- | --- | --- | --- | --- |
|  |  | Beta (95% CI), p-value | Beta (95% CI), p-value | Beta (95% CI), p-value | Beta (95% CI), p-value |
| **Self-reported poor vs. better vision** | Model 3a | -0.34 (-0.66, -0.01), p=0.044* | -0.06 (-0.1, -0.02), p=0.003* | -0.02 (-0.04, 0), p=0.074 | -1.16 (-5.38, 3.07), p=0.592 |
|  | Model 3b | -0.19 (-0.51, 0.13), p=0.243 | -0.03 (-0.07, 0), p=0.068 | -0.01 (-0.03, 0.01), p=0.301 | 0.16 (-3.91, 4.23), p=0.938 |
|  | Model 4 | -0.14 (-0.46, 0.18), p=0.402 | -0.03 (-0.07, 0), p=0.071 | -0.01 (-0.03, 0.01), p=0.37 | 1.04 (-3.04, 5.12), p=0.618 |
| **LogMAR visual acuity** | Model 3a | -0.52 (-1.57, 0.54), p=0.339 | -0.06 (-0.19, 0.06), p=0.341 | -0.06 (-0.13, 0), p=0.063 | -5.83 (-19.45, 7.79), p=0.402 |
|  | Model 3b | 0.01 (-1.01, 1.04), p=0.979 | 0.01 (-0.11, 0.12), p=0.93 | -0.04 (-0.11, 0.02), p=0.185 | 2.04 (-11.1, 15.17), p=0.761 |
|  | Model 4 | 0.1 (-0.92, 1.13), p=0.844 | 0.01 (-0.11, 0.12), p=0.93 | -0.04 (-0.11, 0.02), p=0.21 | 3.41 (-9.68, 16.49), p=0.61 |
| **Log Contrast Sensitivity^a^** | Model 3a | -1.13 (-1.82, -0.43), p=0.001* | -0.03 (-0.11, 0.05), p=0.474 | -0.03 (-0.07, 0.01), p=0.171 | -21.58 (-30.53, -12.64), p<0.001* |
|  | Model 3b | -0.79 (-1.47, -0.12), p=0.022* | 0.01 (-0.06, 0.09), p=0.733 | -0.02 (-0.07, 0.02), p=0.325 | -16.63 (-25.29, -7.96), p<0.001* |
|  | Model 4 | -0.69 (-1.36, -0.01), p=0.048* ^+^ | 0.01 (-0.06, 0.09), p=0.736 | -0.02 (-0.06, 0.02), p=0.384 | -14.79 (-23.46, -6.13), p<0.001*^+^ |
| **Macular degeneration** | Model 4 | -0.55 (-0.97, -0.13), p=0.01* ^+^ | 0 (-0.05, 0.05), p=0.997 | -0.01 (-0.04, 0.02), p=0.367 | -9.33 (-14.67, -3.98), p<0.001* ^+^ |

*Note*. Model 3a contains multiple vision variables (-log contrast sensitivity, logMAR, and self-reported poor vision). Model 3b contains multiple vision variables (-log contrast sensitivity, logMAR, and self-reported poor vision) and is adjusted for age, gender, race, education, body mass index, smoking status, alcohol consumption, diabetes mellitus, hypertension, heart disease, stroke, CESD-10. Model 4 includes Model 3b vision variables and covariates plus macular degeneration. LogMAR = logarithm of the minimum angle of resolution.

^a^Coefficients are for a 1 unit lower log contrast sensitivity (-LCS).

*P-value is <0.05. ^+^P-value is <0.05 if age is removed from the model.
