# Supplementary material for: The relationship between visual function and physical performance in the Study of Muscle, Mobility and Aging (SOMMA)
Source: PLoS One. 2023 Sep 27;18(9):e0292079. doi: 10.1371/journal.pone.0292079 (PMC10529600; doi:10.1371/journal.pone.0292079)
Supplement: S3 Table — (DOCX) [file pone.0292079.s003.docx]

|  |  | **Expanded SPPB Score** | **Narrow Walk Speed (m/sec)** | **Stair Climb (sec)** | **Four-Square Step Test Time (sec)** |
| --- | --- | --- | --- | --- | --- |
|  |  | Beta (95% CI), p-value | Beta (95% CI), p-value | Beta (95% CI), p-value | Beta (95% CI), p-value |
| **Self-reported poor vs. better vision** | Model 3a | -0.11 (-0.2, -0.01), p=0.031 | -0.05 (-0.1, 0), p=0.032* | 1.68 (0.28, 3.08), p=0.019* | 0.51 (-0.03, 1.04), p=0.064 |
|  | Model 3b | -0.04 (-0.13, 0.04), p=0.317 | -0.02 (-0.07, 0.02), p=0.275 | 0.91 (-0.37, 2.2), p=0.164 | 0.37 (-0.15, 0.89), p=0.163 |
|  | Model 4 | -0.04 (-0.12, 0.05), p=0.42 | -0.02 (-0.07, 0.02), p=0.311 | 0.75 (-0.54, 2.05), p=0.256 | 0.31 (-0.22, 0.83), p=0.251^+^ |
| **LogMAR visual acuity** | Model 3a | -0.28 (-0.59, 0.03), p=0.075 | -0.11 (-0.26, 0.05), p=0.169 | 3.36 (-1.12, 7.84), p=0.142 | 1.23 (-0.49, 2.95), p=0.161 |
|  | Model 3b | -0.08 (-0.36, 0.2), p=0.569 | -0.03 (-0.17, 0.12), p=0.719 | 0.3 (-3.82, 4.42), p=0.886 | 0.71 (-0.96, 2.38), p=0.402^+^ |
|  | Model 4 | -0.07 (-0.35, 0.21), p=0.633 | -0.02 (-0.17, 0.12), p=0.754 | 0.05 (-4.08, 4.17), p=0.982 | 0.54 (-1.14, 2.21), p=0.531^+^ |
| **Log Contrast Sensitivity^a^** | Model 3a | -0.36 (-0.57, -0.16), p<0.001* | -0.09 (-0.2, 0.01), p=0.084 | 4.39 (1.45, 7.34), p=0.004* | 1.36 (0.21, 2.51), p=0.021* |
|  | Model 3b | -0.22 (-0.41, -0.04), p=0.016*^+^ | -0.05 (-0.15, 0.05), p=0.303^+^ | 3.3 (0.57, 6.02), p=0.018*^+^ | 1.01 (-0.1, 2.13), p=0.074^+^ |
|  | Model 4 | -0.21 (-0.39, -0.02), p=0.027*^+^ | -0.05 (-0.15, 0.05), p=0.329 | 3 (0.26, 5.74), p=0.032*^+^ | 0.88 (-0.23, 2), p=0.121^+^ |
| **Macular degeneration** | Model 4 | -0.09 (-0.2, 0.03), p=0.141^+^ | -0.01 (-0.07, 0.04), p=0.635 | 1.59 (-0.11, 3.29), p=0.067^+^ | 0.75 (0.04, 1.46), p=0.039*^+^ |

*Note*. Model 3a contains multiple vision variables (-log contrast sensitivity, logMAR, and self-reported poor vision). Model 3b contains multiple vision variables (-log contrast sensitivity, logMAR, and self-reported poor vision) and is adjusted for age, gender, race, education, body mass index, smoking status, alcohol consumption, diabetes mellitus, hypertension, heart disease, stroke, CESD-10. Model 4 includes Model 3b vision variables and covariates plus macular degeneration. LogMAR = logarithm of the minimum angle of resolution.

^a^Coefficients are for a 1 unit lower log contrast sensitivity (-LCS).

*P-value is <0.05. ^+^P-value is <0.05 if age is removed from the model
