# Supplementary material for: The relationship between visual function and physical performance in the Study of Muscle, Mobility and Aging (SOMMA)
Source: PLoS One. 2023 Sep 27;18(9):e0292079. doi: 10.1371/journal.pone.0292079 (PMC10529600; doi:10.1371/journal.pone.0292079)
Supplement: S4 Table — (DOCX) [file pone.0292079.s004.docx]

|  |  | **Leg extension peak power (40-70% of 1 Repetition Maximum, Watts/kg)** | **Leg extension strength (1-Repetition Maximum, Watts/kg)** | **Grip strength (kg)** |
| --- | --- | --- | --- | --- |
|  |  | Beta (95% CI), p-value | Beta (95% CI), p-value | Beta (95% CI), p-value |
| **Self-reported poor vs. better vision** | Model 3a | -0.3 (-0.63, 0.04), p=0.081 | -10.61 (-22.55, 1.32), p=0.082 | -0.45 (-2.29, 1.4), p=0.635 |
|  | Model 3b | 0.003 (-0.27, 0.27), p=0.983 | -0.46 (-9.68, 8.77), p=0.922 | 1.38 (0.07, 2.68), p=0.039* |
|  | Model 4 | -0.0004 (-0.27, 0.27), p=0.998 | -0.7 (-10.02, 8.63), p=0.884 | 1.41 (0.09, 2.72), p=0.036*^+^ |
| **LogMAR visual acuity** | Model 3a | -1.58 (-2.68, -0.49), p=0.005* | -65.27 (-104.19, -26.36), p=0.001* | -8.1 (-14.04, -2.17), p=0.008* |
|  | Model 3b | -0.42 (-1.3, 0.46), p=0.351^+^ | -24.45 (-54.34, 5.44), p=0.109^+^ | -2.65 (-6.83, 1.54), p=0.216 |
|  | Model 4 | -0.42 (-1.31, 0.46), p=0.349 | -24.77 (-54.77, 5.23), p=0.106 | -2.59 (-6.8, 1.61), p=0.227 |
| **Log Contrast Sensitivity^a^** | Model 3a | -0.32 (-1.04, 0.4), p=0.383 | 8.8 (-16.77, 34.36), p=0.5 | -0.29 (-4.22, 3.63), p=0.883 |
|  | Model 3b | -0.35 (-0.93, 0.23), p=0.237^+^ | 3.83 (-15.86, 23.51), p=0.703 | -1.91 (-4.69, 0.87), p=0.18^+^ |
|  | Model 4 | -0.36 (-0.94, 0.22), p=0.228^+^ | 3.32 (-16.51, 23.15), p=0.743 | -1.86 (-4.66, 0.95), p=0.194 |
| **Macular degeneration** | Model 4 | 0.04 (-0.31, 0.4), p=0.81 | 2.59 (-9.47, 14.64), p=0.674 | -0.28 (-1.99, 1.44), p=0.753 |

^a^Coefficients are for a 1 unit lower log contrast sensitivity (-LCS)

Model 3a contains multiple vision variables (-log contrast sensitivity, logMAR, and self-reported poor vision). Model 3b contains multiple vision variables (-log contrast sensitivity, logMAR, and self-reported poor vision) and is adjusted for age, gender, race, education, body mass index, smoking status, alcohol consumption, diabetes mellitus, hypertension, heart disease, stroke, CESD-10. Model 4 includes Model 3b vision variables and covariates plus macular degeneration. LogMAR = logarithm of the minimum angle of resolution.

*P-value is <0.05. ^+^P-value is <0.05 if age is removed from the model
